# The association between dental caries and physical activity, physical fitness, and background factors among Finnish male conscripts

**DOI:** 10.1007/s10266-022-00717-5

**Published:** 2022-05-25

**Authors:** Mika Huttunen, Antti Kämppi, Aapo Soudunsaari, Jari Päkkilä, Leo Tjäderhane, Marja-Liisa Laitala, Vuokko Anttonen, Pertti Patinen, Tarja Tanner

**Affiliations:** 1 Department of Cariology, Endodontology and Pediatric Dentistry, Research Unit of Oral Health Sciences, University of Oulu, P.O. Box 5281, FI-90014 Oulu, Finland; 2 Centre for Military Medicine, Finnish Defence Forces, P.O. Box 5, 11311 Riihimäki, Finland; 3 Department of Oral and Maxillofacial Diseases, University of Helsinki, P.O. Box 41, 00014 Helsinki, Finland; 4 Department of Mathematical Sciences, University of Oulu, P.O. Box 8000, 90014 Oulu, Finland; 5 MRC, Oulu University Hospital and University of Oulu, P.O. Box 5281, 90014 Oulu, Finland; 6 Helsinki University Hospital, P.O. Box 41, 00014 Helsinki, Finland

**Keywords:** Dental caries, Oral health, Physical activity, Physical fitness, Tobacco

## Abstract

Studies on measured physical fitness and oral health are sparse. The aim of this study was to investigate the associations between self-reported physical activity and measured physical fitness and oral health of young men. The study population consisted of 13,564 Finnish male conscripts who had mandatory clinical oral examinations and physical fitness tests at the beginning of military service in 2011. Finally, around 10,800 conscripts had physical fitness test outcomes available and a total of 8552 conscripts answered a computer-based questionnaire on background factors. Decayed Tooth (DT) and Decayed, Missing, or Filled Tooth (DMFT) indices, outcomes of surveys and fitness tests were used in analyses by cross-tabulation and multivariable logistic regression model (odds ratios [OR] with 95% confidence interval [CI]) were calculated. Regularly exercising conscripts had a reduced need for dental restorative treatment than those reporting no physical activity (*p* < 0.0001). The proportion of participants with sound dentition (DT = 0) increased steadily with increasing physical activity (39.0–59.4%). Good measured physical fitness was a protective factor against increased dental restorative treatment need. A low prevalence of smoking and low use of alcohol and energy drinks were associated with frequent exercise, whereas consumption of sport drinks and snuff use were common among those who exercised frequently. Good measured physical fitness and self-reported physical activity are associated with reduced caries burden. There is a need for information about the harms of tobacco products and the benefits of a healthy diet, even for the increased energy needs of the physically active.

## Introduction

Untreated dental caries in permanent teeth is the most common disease worldwide [1]. Poor oral health in top athletes has been reported since the 1960 s [2, 3]. This may be due to the fact endurance sports potentially reduce salivation, increasing the risk of caries and erosion [4]. Additionally, athletes snack often, likely because they have a higher-than-usual need for extra energy [5]. The frequency and total amount of the intake of free sugars or sugar in food play a significant role in the development of dental caries [6–8]. Poor oral hygiene and physiological changes that occur during sport activities are factors which may cause oral diseases [9]. At the same time, young people who play sports are receptive to health-promoting issues [5]. Elite and professional athletes in the UK are willing to consider behavior change to improve oral health [10]. On the other hand, it has been found opposite results that dental caries was significantly less prevalent among those with sufficient (19.8%) than insufficient (27.8%) physical activity among Spanish adults [11]. Insufficient physical activity may lead to overall lifestyle which includes more television watching. In the study among Chinese adolescent, longer duration of television watching was associated with higher risk of developing dental caries [12]. The reason of the association between TV watching and dental caries is not clear. Maybe it is associated lifestyle to consume more sweetened beverage and snacks while watching [13, 14].

There are studies in which physical activity has been linked to different health behaviors. Snuff use is increasing among athletes [15, 16] and those who exercise actively [16, 17]. Snuff use may also indicate current or former smoking and snuff users are more positive about alcohol use [18]. In Finnish study by Päkkilä et al. (2017), the score ≥ 2900 m in the Cooper test and high physical activity decreased odds for smoking [19]. Also, physical exercise is associated with lower alcohol use for middle and high school students in the US, whereas participation in team sports is associated with binge drinking [20].

In Finland, military service is mandatory for men and voluntary for women under 30 years of age. More than 70% of men in each age cohort perform military service annually. The remainder of the age cohort performs civil service or is exempted from service either temporarily or permanently for medical reasons [21]. During service, the conscripts have obligatory physical tests and health examinations and therefore this provides unique study material on health status, including oral health and behaviors of young men and women in their twenties. The outcome of the fitness tests in the Finnish Defence Forces show that body weight has increased, and the physical fitness level has declined among conscripts over the past 20–35 years [22].

There are plenty of studies regarding the oral health of elite athletes but recent literature on the association of physical fitness on oral health among age cohort is sparse.

The aim of this cross-sectional study was to investigate the associations between the burden of dental caries, physical fitness, physical activity, and use of tobacco, alcohol, and energy, soft, and sport drinks among young Finnish male age cohort. We hypothesized that poor physical fitness and low physical activity are associated with unhealthy behaviors and high need and history of dental restorative treatment.

## Materials and methods

### Study population

This cross-sectional study was conducted in 20 garrisons (of a total 24) of the Finnish Defence Forces in January and July 2011. A representative sample of the entire male age cohort born in the beginning of 90 was achieved by examine all conscripts in 15 garrisons and every fifth conscript in alphabetical order in the five largest garrisons. Oral health screening was performed as a part of the obligatory general health examination during the conscripts’ first week in military service. The sample size estimation was not done. Need for dental restorative treatment of each conscript was recorded according to the 1997 criteria for epidemiological studies by the World Health Organization [23] and following the protocol of Finnish Defence Forces. Examinations were performed using the dental unit light, a probe and an oral mirror. All oral examinations were conducted by 15 calibrated dentists, who were trained and calibrated at two full-day sessions before research days in January and July 2011. In these sessions, all examiners were given similar knowledge about the signs of activity and the depth of caries lesions. To practice and test their diagnostic skills, examiners settled the treatment need for 30 photographed and radiographed, extracted teeth with a variety of caries lesions using the WHO criteria. Inter-examiner agreement on treatment need in vitro was ICC = 0.733 (before January’s survey) and ICC = 0.717 (before July’s surveys). Intra-examiner agreement on treatment need was ICC = 0.717. The protocol and reliability of the clinical examinations have been described in more detail previously. [17, 24]. Individual background factors and health behaviors were investigated, including physical activity and dietary and oral hygiene habits. Although the answering the questionnaire was voluntary, all conscripts provided responses. When answering, the conscripts gave permission to use their personal military and health records.

### Description of decayed tooth (DT) and decayed, missing, or filled tooth (DMFT) value

The tooth was recorded as decayed (DT) if a dental caries lesion was detected on any surface of a tooth. If the tooth was either decayed, missing, or filled (DMFT) it had a DMFT value of 1, which represents caries history of the dentition. The DT and DMFT values varied between 0 and 28, as the third molars (wisdom teeth) were excluded from the analyses.

### Variables

The material consisted of a clinical oral health examination, a questionnaire on oral health-related issues, and fitness tests conducted in 2011 for conscripts. DT and DMFT indices were used as indicators of dental health. The responses to the following questions from the questionnaire were used: Do you use snuff? (never or hardly ever/every day or almost every day/occasionally); Do you smoke? (no/1–5 cigarettes daily/10–20 cigarettes daily/ > 20 cigarettes daily); Do you consume alcohol? (no/less than once a month/about once a month/about every other week / about every week/more than once a week); Do you use energy drinks/sports drinks? (never or hardly ever/every day or almost every da /occasionally). Conscripts were also asked How often do you do physical exercise? (never, 1–2 times a month, 1–2 times a week, 3–4 times a week, or more than 5 times a week).

### Categorization of physical fitness test results

Measured physical fitness tests, Cooper test (meters ran in 12 min), push-ups (repetitions in 60 s), and sit-ups (repetitions in 60 s) were categorized as follows: Cooper test (< 2200 m = poor; 2200 m–2799 m = acceptable; 2800 m–3099 m = good; and > 3099 m = excellent), push-ups (< 24 = poor; 24–37 = acceptable; 38–43 = good; and > 43 = excellent) and sit-ups (< 29 = poor; 29–42 = acceptable; 43–48 = good; and > 48 = excellent) [21]. These variables were also dichotomized for logistic regression models. If the Cooper test result was > 2799 m, it was considered good as well as > 37 and > 42 repetitions in push-ups and sit-ups, respectively; otherwise results were considered as poor.

### Categorization of self-reported results

For cross-tabulations, self-reported current amount of exercise was classified as not at all, infrequent (< 2 times a month), weekly (1–2 times a week), frequent (3–4 times a week), and almost daily (> 4 times a week). For the logistic regression model, variables were also categorized in the following three classes: < 1 time a week, 1–4 times a week, > 4 times a week. For the logistic regression model, other self-reported factors were dichotomized as follows: snuff use and smoking; those not smoking at all and all others; and those using snuff at least occasionally during the week and all others. Alcohol use was dichotomized as less than every other week and more often than every other week. The use of energy- and sport drinks was dichotomized as yes or no.

### Categorization of caries experience

For cross-tabulations, DT and DMFT values were categorized as 0, 1–3, and > 3. For the regression model, DT value was dichotomized as DT ≤ 0 or > 0.

### Statistical considerations

Cross-tabulation, *χ*^2^ tests, and multivariable logistic regression model were used in the analyses and odds ratios (OR) with 95% confidence intervals (CI) were calculated. Differences between the groups were considered statistically significant at *p*-values < 0.05. Post hoc testing with Bonferroni repair has been used to find divergent groups in cross-tabulation and *χ*^2^ tests. ROC curve was drawn, and AUC value was calculated to analyze sensitivity and specificity of logistic regression model. Analyses were performed using SPSS version 25.0 (SPSS, Chicago, Illinois, USA) and R version 4.0.2 (R Core Team (2020). R: a language and environment for statistical computing. R Foundation for Statistical Computing, Vienna, Austria).

### Ethical considerations

The index data were collected from the archives of the records of the Finnish Defence Forces with their permission. The research plan was evaluated by the Ethics Committee of the Northern Ostrobothnia Hospital District and a positive statement was issued on the 29 March 2010 (EETTMK: 27/2010 71§). The Center for Military Medicine and the Defence Forces Staff gave the permission for the study on the 23 June 2010 (AG14218). The conscripts gave their consent to use their personal data by answering the computer-based questionnaire.

## Results

Study group consisted of 13,564 men born in 1990, 1991, or 1992 (mean age 19.6 years) and more than 10,800 of them was the results of physical fitness tests of Finnish Defence Forces available. Number available was slightly differ between the physical fitness tests (Cooper’s test = 10,808; push-ups = 10,882; sit-ups = 10,949). The reasons for the missing data can only be speculated. Perhaps, the conscripts in question were inhibited at the time arranged for physical fitness tests and in the big organization, no additional performance shifts could be arranged. In connection with the oral screening, 8522 male conscripts answered a computer-based questionnaire because of tight schedule just part of the conscripts has time to answered questionnaire.

According to the DT and DMFT values, oral health was significantly better in regularly exercising conscripts than those who did not exercise at all (Table 1). The greatest difference was observed with conscripts who exercised > 4 times a week. The proportion of conscripts with DT = 0 increased steadily as physical activity increased (39.0–59.4%). Based on post hoc tests, further evidence has got that the proportion of DT > 3 in those with little exercise is higher than those with high levels of exercise and vice versa (data not shown).

**Table 1:** Proportions of the conscripts as for categorized amount of self-reported physical activity in association with restorative treatment need (DT), as well as present and past caries experience (DMFT)

	How frequently do you exercise (times)?					*p*
	Not at all	1–2 / m	1–2 / w	3–4 / w	> 4 /w	
DT (%/*n*)						< 0.001
0	39.0/176	47.2/666	52.5/1,678	56.1/1,446	59.4/545	
1–3	38.4/173	34.7/489	32.7/1,045	32.7/843	32.3/296	
> 3	22.6/102	18.1/255	14.8/473	11.2/289	8.3/76	
Total	100	100	100	100	100	
DMFT (%/*n*)						< 0.001
0	14.2/64	16.9/238	19.6/626	21.8/562	22.4/205	
1–3	29.0/131	29.8/420	33.1/1,058	35.6/918	39.5/362	
> 3	56.8/256	53.3/752	47.3/1,512	42.6/1,098	38.2/350	
Total %/*n*	100/451	100/1,410	100/3,196	100/2,578	100/917	

*n* = 8552

Similarly, the proportion of conscripts with more than three lesions needing dental restorative treatment decreased steadily as the frequency of exercise increased (22.6–8.3%). The same phenomenon was observed for the DMFT index (Table 1).

A low prevalence of smoking and low use of alcohol and energy drinks were associated with frequent exercise. The converse was true for the use of snuff and sports drinks, which increased with increasing exercise frequency. Energy drinks were mostly consumed by those who exercised infrequently, whereas consumption of sport drinks was common among those who exercised frequently (Table 2).

**Table 2:** Amount of self-reported exercise by conscripts, smoking, snuff, alcohol, energy drink, and sport drink use by section

	How frequently do you exercise or play/m (month) or /w (week)?					*p*
	Not at all	1–2 / m	1–2 / w	3–4 / w	> 4 /w	
Do you smoke? (%/*n*)						< 0.001
I do not	40.6/183	44.5/627	55.5/1774	70.7/1822	84.8/778	
1–5 cigarettes a day	10.9/49	13.3/187	14.0/447	12.9/333	8.5/78	
10–20 cigarettes a day	39.7/179	37.8/533	28.1/898	15.0/387	5.8/53	
> 20 cigarettes a day	8.9/40	4.5/63	2.4/77	1.4/36	0.9/8	
Total %/*n*	100/451	100/1410	100/3196	100/2578	100/917	
Do you use snuff? (%/*n*)						< 0.001
Never or almost never	86.0/388	84.4/1,190	83.7/2,675	77.9/2,008	72.2/662	
Occasionally during the week	7.3/33	10.1/142	9.4/300	10.9/281	11.1/102	
Every day or almost every day	6.7/30	5.5/78	6.9/221	11.2/289	16.7/153	
Total %/*n*	100/451	100/1,410	100/3,196	100/2,578	100/917	
Do you drink alcohol? (%/n)						< 0.001
I do not	10.9/49	7.4/104	7.9/252	9.8/253	8.9/82	
Less than once a month	12.0/54	11.1/157	9.7/310	8.9/229	15.7/144	
Approximately once a month	14.9/67	19.5/275	21.9/700	24.2/623	29.6/271	
About every other week	18.8/85	29.9/422	34.7/1,109	35.6/918	31.7/291	
Approximately once a week	29.0/131	24.8/350	20.9/668	19.3/498	11.5/105	
More than once a week	14.4/65	7.2/102	4.9/157	2.2/57	2.6/24	
Total %/*n*	100//451	100/1,410	100/3,196	100/2,578	100/917	
Do you use energy drinks? (%/*n*)						< 0.001
Never or not at all	45.0/203	46.2/651	49.5/1,582	54.2/1,397	58.3/535	
Occasionally during the week	41.7/188	43.9/619	43.9/1,403	40.8/1,052	36.4/334	
Every day or almost every day	13.3/60	9.9/140	6.6/211	5.0/129	5.3/48	
Total %/*n*	100/451	100/1,410	100/3,196	100/2,578	100/917	
Do you use sport drinks? (%/*n*)						< 0.001
Never or not at all	92.5/417	89.8/1,266	83.2/2,659	69.3/1,787	48.0/440	
Occasionally during the week	5.5/25	9.5/134	15.9/508	26.3/678	38.9/357	
Every day or almost every day	2.0/9	0.7/10	0.9/29	4.4/113	13.1/120	
Total %/*n*	100/451	100/1,410	100/3,196	100/2578	100/917	

Proportions of the conscripts as for categorized amount of self-reported physical activity in association with smoking, use of snuff, and consumption of alcohol, energy and sport drinks. *n* = 8552

**Fig. 1:**
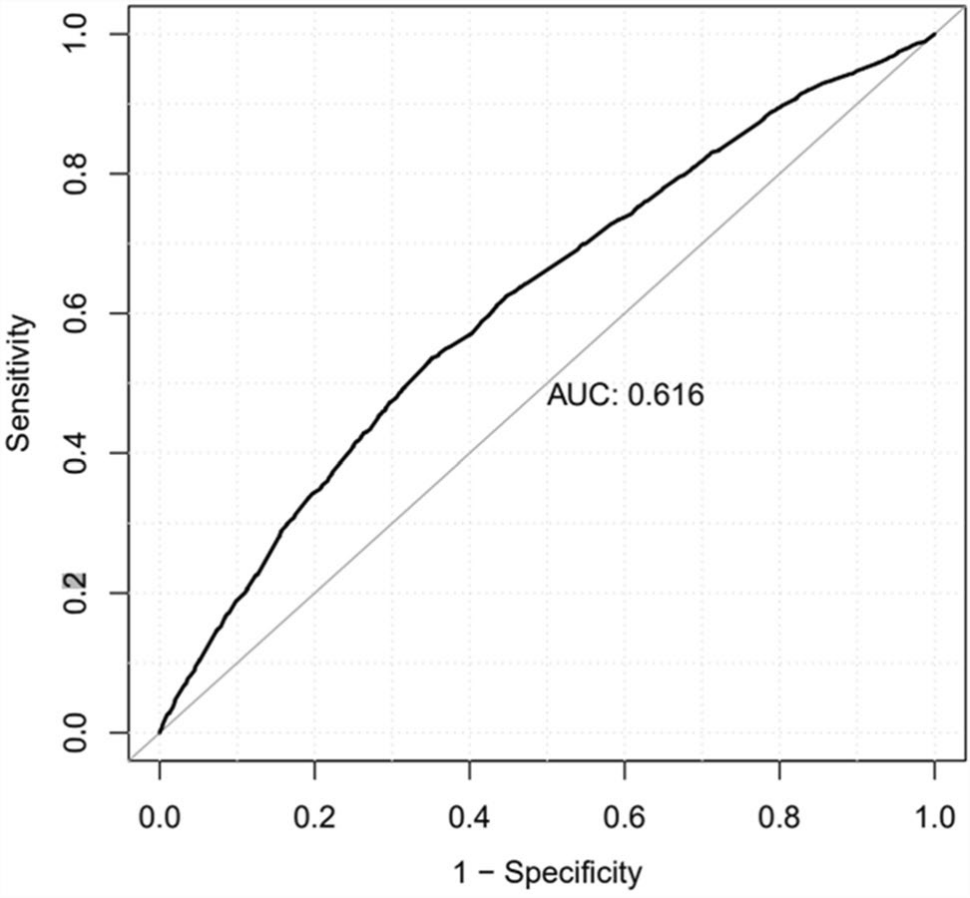
ROC curve measuring the sensitivity and specificity of logistic regression model with DT > 0 as dependent variable and categorized health behaviors, self-reported physical activity and outcomes of physical fitness tests explaining (independent) variables

Table 3 shows the results of physical fitness tests and categorized DT and DMFT values. DT = 0 and DMFT = 0 were most common among conscripts who achieved the highest scores (> 43 push-ups, > 48 sit-ups or > 3099 m from the Cooper test) (Table 3.) However, in the regression model, a low Cooper test score did not increase the odds for dental restorative treatment need (DT > 0), in contrast to smoking (OR = 1.91) or consumption of energy drinks (OR = 1.24) or low results of push-ups (OR = 1.14) and sit-ups (OR = 1.34). On the other hand, alcohol use every other week or more often was associated with no need of restorative treatment need (DT = 0) (Table 4). The AUC value of logistic regression model was 0.616 (Fig. 1).

**Table 3:** Proportions of the conscripts as for categorized physical fitness test outcomes in association with restorative treatment need (DT), as well as present and past caries experience (DMFT)

	Push-ups					Sit-ups					Cooper				
	< 24	24–37	38–43	> 43	*p*	< 29	29–42	43–48	> 48	*p*	< 2200 m	2200–2799 m	2800–3099 m	> 3099 m	*p*
DT (%/*n*)					< 0.001					< 0.001					< 0.001
0	51.6/ 1.701	54.8/ 2.162	57.1/ 1.034	62.2/ 1.139		49.8/ 1.131	54.2/ 2.878	59.5/ 1.072	63.0/ 986		48.7/ 1.086	55.7/ 3.603	59.7/ 1.069	64.1/ 205	
1–3	32.2/ 1.061	32.2/ 1.270	31.7/ 574	30.1/ 551		33.5/ 761	32.0/ 1,700	31.0/ 558	29.3/ 459		34.5/ 769	31.5/ 2,037	31.3/ 560	29.1/ 93	
> 3	16.2/ 534	13.0/ 513	11.2/ 202	7.7/ 141		16.7/ 379	13.8/ 733	9.5/ 171	7.7/ 121		16.8/ 375	12.8/ 828	9.0/ 161	6.9/ 22	
Total (*n*)	3.296	3.945	1.810	1.831		2.271	5.311	1.801	1.566		2.230	6.468	1.790	320	
DMFT (%/*n*)					< 0.001					< 0.001					< 0.001
0	19.9/ 656	20.9/ 824	22.6/ 409	27.0/ 494		18.5/ 420	21.0/ 1.115	24.7/ 445	26. 5/ 415		18.4/ 410	21.4/ 1.384	26.1/ 467	30.3/ 97	
1–3	31.1/ 1.025	34.9/ 1.377	34.0/ 615	34.4/ 630		30.7/ 697	33.7/ 1.790	35.2/ 643	35.8/ 561		31.2/ 696	33.5/ 2.167	35.6/ 637	37.8/ 121	
> 3	49.0/ 1.615	44.2/ 1.744	43.4/ 786	38.6/ 707		50.8/ 1.154	45.3/ 2.406	40.1/ 722	37.7/ 590		50.4/ 1124	45.1/ 2.917	38.3/ 686	31.9/ 102	
Total %	100	100	100	100		100	100	100	100		100	100	100	100	
*n*	3.296	3.945	1.810	1.831		2.271	5.311	1.801	1.566		2.230	6.468	1.790	320	

*n* = 10,882 (Push-ups), *n* = 10,949 (Sit-ups), *n* = 10,808 (Cooper)

**Table 4:** Logistic regression analysis with DT > 0 as dependent variable and categorized health behaviors, self-reported physical activity and outcomes of physical fitness tests explaining (independent) variables

Explanatory variable		DT > 0					
		OR		95% CI	SE	*z*	*P*-value
Do you smoke							
No		1					
Yes		1.91		1.71, 2.13	0.06	11.59	< 0.001
Do you use snuff							
No		1					
Yes		1.05		0.92, 1.19	0.07	0.67	0.50
Do you consume alcohol							
< every other week		1					
≥ every other week		0.79		0.71, 0.88	0.05	− 4.40	< 0.001
Do you use energy drinks							
No		1					
Yes		1.24		1.12, 1.37	0.05	4.01	< 0.001
Do you use sport drinks							
No		1					
Yes		1.06		0.93, 1.20	0.06	− 2.10	0.40
Push-ups							
Good		1					
Poor		1.14		1.01, 1.29	0.06	− 2.10	0.04
Sit-ups							
Good		1					
Poor		1.34		1.18, 1.52	0.07	− 4.46	< 0.001
Cooper test							
Good		1					
Poor		0.91		0.79, 1.04	0.07	1.40	0.175
Current amount of exercise							
Less than once/week		1					
1–4 times a week	0.89		0.78, 1.01		0.07	− 1.83	0.067
> 4 times a week	0.90		0.72, 1.12		0.11	− 0.94	0.346

## Discussion

The main findings of the present study were that good physical fitness and high physical activity were associated with lower caries prevalence (DT) and history (DMFT). In the regression model, poor results from sit-ups and push-ups, daily smoking, and use of energy drinks increased the odds for caries incidence (DT > 0) whereas alcohol use every other week or often decrease the odds for restorative treatment need. On the other hand, physically active conscripts used more snuff and sport drinks than their less active counterparts, which means that the study hypothesis was only partially correct.

The results of the association between physical activity and dental caries are inconsistent with previous literature. Different outcomes between the studies may be explained by different research settings, such as differences in the assessment of physical activity and of dental caries (self-reported vs clinical examination). In the Spanish study [10], self-reported high physical activity was associated with self-reported dental caries. However, the association disappeared in an adjusted (sex, age, marital status, education, obesity, smoking, alcohol) logistic regression model. This can be explained by mean age differences (20–48.5 years) and the fact that in the present study caries experience was not self-reported. In a systematic review, Needleman et al. (2015) observed that poor oral health in top athletes has been reported since the 1960 s [2]. Nutritional intake including usual diet, use of sport drinks and supplements, and dehydration and drying of the oral cavity during sports activity are some of the oral health challenges for elite athletes [2]. In the present study, the population represents one Finnish male age cohort, not just athletes, which may explain the differences between the studies. However, the present findings of high consumption of sport drinks among those with high physical activity is consistent with previous studies [14, 25].

An interesting finding was that good muscular strength (sit-ups, push-ups) and Cooper test were associated with no caries experience (DT = 0). This outcome was also evident in the regression analysis but not for excellent performance in the Cooper test (> 2799 m). To our knowledge, there are no previous studies about the association between measured physical fitness and dental caries. The reasons for differences between different physical fitness tests concerning caries risk can only be speculated. It seems that a conscript with good results in the push-ups or sit-ups test did not necessarily achieve good results in the Cooper test. A conscript with good results in the Cooper test possibly had more experience in endurance sports, which potentially reduce saliva secretion [16] and lead to a greater-than-usual need for extra energy. This additional need may be filled with carbohydrate-containing sport drinks and gels [2]. Previous sport activity type of the conscripts was not investigated in the present study. Frese et al. [4] investigated the effect of endurance training on dental caries, tooth erosion, and saliva and observed that endurance athletes have a significantly higher risk for erosive tooth wear. There were no differences of caries prevalence and saliva parameters between the athletes and control group [16]. Erosive tooth wear was not studied here. It can be speculated bilateral pathway of association between physical fitness and activity and dental caries, i.e., physical fitness may affect dental caries and dental caries may affect physical fitness. There are a lot of behavioral issues that can positively affect both physical fitness and oral health. Regular rhythm of life and healthy eating habits may be associated with physically active life and known to be good for oral health too. Gallagher et al. (2019) found that professional athletes known to be willing to consider behavior change to improve oral health [10].

Approximately, 90% of the study population consumed alcohol at least occasionally. Moderate alcohol consumption (less than once a week) did not appear to be associated with physical activity, consistent with a previous study [26]. Consuming alcohol at least once a week already seems to decrease the amount of self-reported exercise. It would have been more valuable to investigate the total amount of alcohol use instead of frequency of use. That was not possible in the present study.

In Finland, smoking has decreased among young people and snuff use has increased, especially among athletes and those who exercise frequently [15–17]. This is consistent with the results in the present study, where daily snuff use was almost three times more common among those who exercised > 4 times a week than among those who did not exercise at all. Again, among physically active persons (exercise > 4 times a week), non-smoking was two times more common than among conscripts who did not exercise at all. Snuff use may be perceived as a healthy alternative to cigarette smoking. Snuff and other smokeless tobacco products are highly addictive due to the high amount of nicotine [27, 28]. Nicotine addiction is considered a disease requiring treatment [29]. In addition, snuff use among men is associated with increased all-cause mortality, cardiovascular mortality, and with possibly increased cancer mortality [30].

The strength of this study is the multifaceted approach to lifestyles that may affect or be associated with oral health. This study included a large cross-section of different socio-economic groups, as mandatory military service is a unique environment that brings together these different groups. However, those with the most serious general diseases are exempted from service and therefore practically all participants were healthy; thus, the effect of chronic diseases could not be included in the analyses. The participants in the study were all males; the results cannot be generalized to young females. Information of health habits, such as smoking and exercise activity, was self-reported. This may have caused some bias in the outcome, as it is possible that respondents over- or underestimated their health behaviors. However, with such a large sample size, this bias is not anticipated to have a considerable effect on the results. In addition, some other common risk factors for dental caries such as the use of fluoride products, snacking and socio-economic status were not investigated in the study. So, the further studies are needed to investigate reasons behind the oral health differences between different physical activity and physical fitness groups. There are also some limitations regarding study design. Cross-sectional study offers a snapshot of study group and causal relationships cannot describe.

It can be concluded that good physical fitness and high physical activity appear to be associated with a lower need for dental restorative treatment. Maybe young men who are interested in physical training, also are more interested about their oral health. Recommending a healthy lifestyle may help young men even more holistic. Otherwise, use of snuff and sport drinks is common among men with high physical activity. To cover increased energy needs during exercise and free time, healthy dietary options that are not detrimental to oral health should be recommended to physically active persons. Support for cessation of tobacco products should also be provided.

## References

[1] Kassebaum NJ, Smith AGC, Bernabé E, Fleming TD, Reynolds AE, Vos T, Murray CJL, Marcenes W (2017). Global, regional, and national prevalence, incidence, and disability-adjusted life years for oral conditions for 195 countries, 1990–2015: a systematic analysis for the global burden of diseases, injuries, and risk factors. J Dent Res.

[2] Needleman I, Ashley P, Fine P, Haddad F, Loosemore M, Medici A, Donos N, Newton T, Someren K, Moazzez R, Jaques R, Hunter G, Khan K, Shimmin M, Brewer J, Meehan L, Mills S, Porter S (2015). Oral health, and elite sport performance. Br J Sports Med.

[3] Ashley P, Di Iorio A, Cole E, Tanday A, Needleman I (2015). Oral health of elite athletes and association with performance: a systemic review. Br J Sports Med.

[4] Frese C, Frese F, Kuhlmann S, Saure D, Reljic D, Staehle HJ, Wolff D (2015). Effect of endurance training on dental erosion, caries, and saliva. Scand J Med Sci Sports.

[5] Anttonen V, Kemppainen A, Niinimaa A, Pesonen P, Tjäderhane L, Laitinen J (2014). Dietary and oral hygiene habits of active athletes and adolescents attending ordinary junior high schools. Int J Paediatr Dent.

[6] Akarslan ZZ, Sadik B, Sadik E, Erten H (2008). Dietary habits and oral health related behaviors in relation to DMFT indexes of a group of young adult patients attending a dental school. Med Oral Patol Oral Cir Bucal.

[7] Sheiham A, James WP (2014). A reappraisal of the quantitative relationship between sugar intake and dental caries: the need for new criteria for developing goals for sugar intake. BMC Public Health.

[8] Bernabé E, Vehkalahti; MM; Sheiham, A; Lundqvist, A; Suominen, AL.  (2016). The shape of the dose-response relationship between sugars and caries in adults. J Dent Res.

[9] Tripodi D, Cosi A, Fulco D, D’Ercole S (2021). The impact of sport training and oral health in athletes. Dent J.

[10] Gallegher J, Ashley P, Petrie A, Needleman I (2019). Oral health-related behaviours reported by elite and professional athletes. Br Dent J.

[11] Guillermo F, Lopez S, Smith L, Koyanagi A, Grabovac I, Lin Yang, Veronese N, Shin JI, Loosemore M, Jacob L (2020). Associations between self-reported physical activity and oral health: a cross-sectional analysis in 17,777 Spanish adults. Br Dent J.

[12] Zeng X, Sheiham A, Sabbah W (2014). The association between dental caries and television viewing among Chinese adolescents in Guangxi China. BMC Oral Health.

[13] Ghimire N, Rao A (2013). Comparative evaluation of the influence of television advertisements on children and caries prevalence. Glob Health action.

[14] Kelly B, Halford JC, Boyland EJ, Chapman K, Bautista-Castaño I, Berg C, Caroli M, Cook B, Coutinho JG, Effertz T (2010). Television food advertising to children: a global perspective. Am J Public Health.

[15] Mattila VM, Raisamo S, Pihlajamäki H, Mäntysaari M, Rimpelä A (2012). Sports activity and the use of cigarettes and snus among young males in Finland in 1999–2010. BMC Public Health.

[16] Henninger S, Fischer R, Cornuz J, Studer J, Gmel G (2015). Physical activity and snus: is there a link?. Int J Environ Res Public Health.

[17] Tanner T, Kämppi A, Päkkilä J, Järvelin M-R, Patinen P, Tjäderhane L, Anttonen V (2014). Association of smoking and snuffing with dental caries occurrence in a young male population in Finland: a cross-sectional study. Acta Odontol Scand.

[18] Tseveenjav B, Pesonen P, Virtanen JI (2015). Use of snus, its association with smoking and alcohol consumption, and related attitudes among adolescents: the Finnish national school health promotion study. Tob Induc Dis.

[19] Päkkilä J, Anttonen V, Patinen P, Nyman K, Valkeapää K, Birkhed D, Tjäderhane L, Tanner T (2017). Profiling of smokers and snuffers among young Finnish men - cross-sectional epidemiological study. Acta Odontol Scand.

[20] Terry-McElrath YM, O’Malley PM, Johnston LD (2011). Exercise, and substance use among American youth, 1991–2009. Am J Prev Med.

[21] Lehesjoki, M. Interruption of conscript service for health reasons, National Defense College, Publication Series 1: Studies No. 27, 19992018, National Defence University, PhD thesis, Helsinki, 2018. (In Finnish with English abstract). https://www.doria.fi/bitstream/handle/10024/157248/Lehesjoki_final_verkkoversio.pdf?sequence=1&isAllowed=y. Accessed on Apr 2021).

[22] Defence Forces database (in Finnish). VARUSMIESPALVELUKSEN ALOITTAVIEN MIESTEN FYYSISEN TOIMINTAKYVYN TILASTOT 1975–2020. https://puolustusvoimat.fi/documents/2035479/2042680/VM+kuntotilastot+2020.pdf/b3235be5-ed79-6054-c98a-f6812cc0446c/VM+kuntotilastot+2020.pdf?t=1605694377563. Accessed 9 Jan 2021

[23] World Health Organization. Oral health surveys-basic methods. 4th ed. Geneva: World Health Organization; 1997. p 39–44 https://apps.who.int/iris/bitstream/handle/10665/41905/9241544937.pdf?sequence=1&isAllowed=y. Accessed 3 June 2021

[24] Tanner T, Kämppi A, Päkkilä J, Patinen P, Karjalainen K, Rosberg J, Tjäderhane L, Anttonen V (2013). Prevalence and polarization of dental caries among young, healthy adults – cross-sectional epidemiological study. Acta Odontol Scand.

[25] Bryant S, McLaughlin K, Morgaine K, Drummond B (2011). Elite athletes and oral health. Int J Sports Med.

[26] Paavola M, Vartiainen E, Haukkala A (2004). Smoking, alcohol use and physical activity: a 13-year longitudinal study ranging from adolescence into adulthood. J Adolesc Health.

[27] Benowitz NL (2010). Nicotineaaddiction. N Engl J Med.

[28] Fagerstrom K (2018). A Comparison of dependence across different types of nicotine containing products and coffee. Int J Environ Res Public Health.

[29] Prevention and treatment of tobacco and nicotine addiction. Current Care Guidelines. Working group set up by the Finnish Medical Society Duodecim and the Finnish Association for General Practice. Helsinki: The Finnish Medical Society Duodecim, 2018 (referred May 7, 2020). https://www.kaypahoito.fi/hoi40020. Accessed 3 June 2021

[30] Byhamre ML, Araghi M, Alfredsson L, Bellocco R, Engström G, Eriksson M, Galanti MR, Jansson JH, Lager A, Lundberg M, Östergren PO, Pedersen NL, Trolle Lagerros Y, Ye W, Wennberg P, Magnusson C (2021). Swedish snus use is associated with mortality: a pooled analysis of eight prospective studies. Int J Epidemiol.

